# Occupational Exposure to Needle Stick Injuries and Hepatitis B Vaccination Coverage Among Clinical Laboratory Staff in Sana’a, Yemen: Cross-Sectional Study

**DOI:** 10.2196/15812

**Published:** 2020-03-31

**Authors:** Nabil Al-Abhar, Ghuzlan Saeed Moghram, Eshrak Abdulmalek Al-Gunaid, Abdulwahed Al Serouri, Yousef Khader

**Affiliations:** 1 Field Epidemiology Training Program Sana'a Yemen; 2 National Center of Public Health Laboratories Sana'a Yemen; 3 Al Thawra Hospital Sana'a Yemen; 4 Jordan Field Epidemiology Training Program Jordan Ministry of Health Amman Jordan

**Keywords:** injury, hepatitis B, vaccination, biosafety, laboratory staff, Yemen

## Abstract

**Background:**

Laboratory staff handling blood or biological samples are at risk for accidental injury or exposure to blood-borne pathogens. Hepatitis B virus (HBV) vaccinations for laboratory staff can minimize these risks.

**Objective:**

The aims of this study were to determine the prevalence of occupational exposure to needle stick injuries (NSIs) and assess HBV vaccination coverage among clinical laboratory staff in Sana’a, Yemen.

**Methods:**

A cross-sectional survey was conducted among clinical laboratory staff who were involved in handling and processing laboratory samples at the main public and private clinical laboratories in Sana’a. Data collection was done using a semistructured questionnaire. The questionnaire was divided into 3 parts. Part 1 included information on sociodemographic characteristics of participants. Part 2 included information on the availability of the personal protective equipment in the laboratories, such as lab coats and gloves. Part 3 included questions about the history of injury during work in the laboratory and the vaccination status for HBV.

**Results:**

A total of 219/362 (60%) participants had been accidentally injured while working in the laboratory. Of those, 14.6% (32/219) had been injured during the last 3 months preceding the data collection. Receiving the biosafety manual was significantly associated with lower risk of injury. Out of those who were injured, 54.8% (120/219) had received first aid. About three-quarters of respondents reported that they had been vaccinated against HBV. The vaccination against HBV was significantly higher among laboratory staff who were working at private laboratories (*P*=.01), who had postgraduate degrees (*P*=.005), and who received the biosafety manual (*P*=.03).

**Conclusions:**

Occupational exposure to NSI is still a major problem among laboratory staff in public and private laboratories in Sana’a, Yemen. The high incidence of injuries among laboratory staff and the low rate of receiving first aid in laboratories combined with low vaccination coverage indicates that all laboratory staff are at risk of exposure to HBV. Therefore, strengthening supervision, legalizing HBV vaccinations for all laboratory staff, and optimizing laboratory practices regarding the management of sharps can minimize risks and prerequisites in Yemen.

## Introduction

Laboratory staff handling blood or biological samples are at risk for accidental injury or exposure to blood-borne pathogens [1,2]. This may occur through exposure to aerosols, spills and splashes, accidental needle stick injuries (NSIs), cuts from sharp objects and broken glass, oral pipetting, and centrifuge accidents [3,4].

The World Health Organization reported that about three million health care workers worldwide experience percutaneous exposure to blood-borne viruses. Consequently, 2.5% of HIV cases and 40% of Hepatitis B and C cases occurred among health workers worldwide [3,5]. Furthermore, different NSI prevalence were reported among laboratory staff from Kenya (25%), Saudi Arabia (14%), and Iran (2.3%) [6-8].

Laboratory staff are at high risk of blood-borne viruses including HIV and hepatitis B and C because of the limited vaccination of hepatitis B virus (HBV) among health care workers, the lack of personal protective equipment, and unsafe work practices such as improper management of sharp waste [9-11].

There is a scarcity of data in Yemen about occupational exposure to NSIs and HBV vaccination coverage among laboratory staff. One study reported that 55% of staff had been injured during their work in the laboratory, with NSIs being the commonest injury, and only 47% of staff had been vaccinated against HBV [12]. This study aimed to determine prevalence of occupational exposure to NSIs and assess HBV vaccination coverage among clinical laboratory staff in public and private laboratories in Sana’a, Yemen.

## Methods

### Study Design and Study Population

A descriptive cross-sectional study was conducted among all laboratory staff who were involved in processing laboratory samples in the main public and private laboratories in Sana’a. The study included those who were working in the National Center of Public Health Laboratories as well as three of the main public laboratories (Al-Thawra, Al-Jomhory, and Al-Kuwait) and three of the main private laboratories (Saudi Germany, University of Science and Technology, and Azal). Staff who were not involved in processing laboratory samples, such as administrative staff, were excluded.

### Data Collection and the Study Questionnaire

Data was collected between September 1 and October 31, 2015, using a self-administered semistructured questionnaire. The quality control officers at each laboratory were trained to distribute the questionnaires to the participants, collect the necessary data, and review the filled questionnaires on the spot. Ethical approval was obtained from the National Committee for Medical and Health Research at the Ministry of Public Health and Population. Informed consent was obtained from all participants.

The questionnaire was pilot tested on 10 respondents, who were not included in this study, and necessary changes were made. The questionnaire was developed based on the available standard guidelines and practices and the reviewed literature [3,6,8-10], as well as feedback from some experts in the field. The questionnaire was divided into 3 parts. Part 1 included information on sociodemographic characteristics of participants. Part 2 included information on the availability of the personal protective equipment in the laboratories, such as lab coats and gloves. Part 3 included questions about the history of injury during laboratory work and the vaccination status for HBV.

### Statistical Analysis

Data was analyzed using SPSS version 18 (SPSS Inc, Chicago, IL). Data was analyzed using frequencies and percentages. The differences between proportions according to studied characteristics were tested using the chi-square test. A *P*<.05 was considered statistically significant.

## Results

Of 385 laboratory staff, 362 (292 from public laboratories and 70 from private laboratories) completed the study questionnaire with a response rate of 94.0%. Table 1 shows the respondents’ sociodemographic characteristics. About half of the respondents were 30 to 39 years of age. More than two-thirds (298/362, 82.3%) had received a bachelor’s degree, and 47.5% (172/362) had more than 10 years of work experience.

A total of 219/362 (60.5%) respondents had been accidentally injured during their work in the laboratory (Table 2). Of those, 32/219 (14.6%) had been injured during the 3 months preceding data collection.

Table 3 shows the availability of personal protective equipment in public and private laboratories. The majority of laboratory staff reported wearing gloves and lab coats with no significant difference between private and public laboratories. Although other personal protective equipment (eg, masks, goggles, safety cabinets, and eye washers) were generally less available; however, private laboratory staff reported significantly higher availability (*P*<.001). Receiving a biosafety manual was the only factor that was significantly associated with lower injury incidence. Out of those who were injured, 120/219 (54.8%) had received first aid. Those who were working at private laboratories and those who had received a biosafety manual and biosafety training were significantly more likely to receive first aid.

About three-quarters of respondents reported that they had been vaccinated (ie, received the recommended 3 doses) against HBV (Table 4). The vaccination against HBV was significantly higher among laboratory staff who were working at private laboratories, those who had postgraduate degrees, and those who received the biosafety manual.

**Table 1:** Sociodemographic characteristics of laboratory workers (N=362).

Variable		Laboratory workers, n (%)
**Gender**		
	Male	178 (49.2)
	Female	184 (50.8)
**Age (year)**		
	20-29	83 (22.7)
	30-39	188 (51.9)
	40-49	70 (19.3)
	50-59	20 (5.5)
	>59	1 (0.3)
**Education**		
	Diploma	64 (17.7)
	Bachelor’s	223 (61.6)
	Higher than Bachelor’s	75 (20.7)
**Work experience (years)**		
	1-4	60 (16.6)
	5-10	130 (35.9)
	11-15	74 (20.4)
	>15	98 (27.1)

**Table 2:** History of injury among laboratory staff during their work, and associated factors (N=362).

Variables		Injured, n (%)	Not injured, n (%)	*P* value
**Type of laboratory**				**.30**
	Public	181 (62.0)	111 (38.0)	
	Private	38 (54.3)	32(45.7)	
**Gender**				**.41**
	Male	112 (62.9)	66 (37.1)	
	Female	107 (58.2)	77 (41.8)	
**Education**				**.45**
	Nonpostgraduate	177 (61.7)	110 (38.3)	
	Postgraduate	42 (56.0)	33 (44.0)	
**Work experience (years)**				**.91**
	1-10	116 (61.1)	74 (38.9)	
	>10	103 (59.9)	69 (40.1)	
**Received biosafety manual**				**.01**
	No	189 (63.6)	108 (36.4)	
	Yes	30 (46.2)	35 (53.8)	
**Received biosafety training**				**.13**
	No	141 (63.8)	80 (36.2)	
	Yes	78 (55.3)	63 (44.7)	

**Table 3:** Availability of personal protective equipment in public and private laboratories.

Personal protective equipment	Total (N=362), n (%)	Public laboratories (n=292), n (%)	Private laboratories (n=70), n (%)	*P* value
Gloves	346 (95.6)	276 (94.5)	70 (100.0)	.09
Lab coats	350 (96.7)	280 (95.9)	70 (100.0)	.18
Masks	89 (24.6)	51 (17.5)	38 (54.3)	<.001
Goggles	28 (7.7)	13 (4.5)	15 (21.4)	<.001
Safety cabinet	122 (33.7)	68 (23.3)	54 (77.1)	<.001
Eye washer	70 (19.3)	38 (13.0)	32 (45.7)	<.001

**Table 4:** Hepatitis B virus vaccination status and associated factors (N=362).

Variables		Not vaccinated, n (%)	Vaccinated, n (%)		*P* value
**Type of laboratory**				**.01**	
	Public	77 (26.4)	215 (73.6)		
	Private	8 (11.4)	62 (88.6)		
**Gender**				**.75**	
	Male	40 (22.5)	138 (77.5)		
	Female	45 (24.5)	139 (75.5)		
**Education**				**.005**	
	Nonpostgraduate	77 (26.8)	210 (73.2)		
	Postgraduate	8 (10.7)	67 (89.3)		
**Work experience (years)**				**.09**	
	1-10	52 (27.4)	138 (72.6)		
	>10	33 (19.2)	139 (80.8)		
**Received biosafety manual**				**.03**	
	No	77 (25.9)	220 (74.1)		
	Yes	8 (12.3)	57 (87.7)		
**Received biosafety training**				**.36**	
	No	56 (25.3)	165 (74.7)		
	Yes	29 (20.6)	112 (79.4)		

## Discussion

Occupational exposure to NSI increases the risk of acquiring serious blood-borne infections among health care workers. Our findings showed that 60% of the laboratory staff had been injured while working in laboratories. Similar prevalence rates were reported from studies in Sana’a and India [12,13]. However, lower rates were reported from other countries including Kenya (25%), Saudi Arabia (14%), and Iran (2.3%) [6-8].

Our findings showed low availability of some key personal protective equipment (eg, masks, goggles, safety cabinets, and eye washers) especially in public laboratories. Injury in the laboratory was significantly less likely among laboratory staff who had received the biosafety manual. This indicates that training on biosafety helped to raise awareness as well as improve attitudes and protective practices [14]. Therefore, training of laboratory staff on biosafety manuals and making personal protective equipment available are crucial to reduce exposure to NSIs and its possible grave consequences. Furthermore, strengthening the biosafety program and policies in laboratories together with enforcing use of personal protective equipment should be a cornerstone for reducing high NSIs in Yemen.

Half of the injured staff had received first aid. A lower percentage (28.8%) was reported in other counties including India [15]. There is a scarcity of data regarding HBV vaccination coverage among laboratory staff. Our study showed that only three-quarters of laboratory staff were vaccinated against HBV. In Saudi Arabia and Libya, studies showed that 97% and 82% were vaccinated against HBV, respectively [6,16]. In a previous study among laboratory staff in three public laboratories in Sana’a, 47% were vaccinated against HBV [12]. The coverage of the HBV vaccine was found to be significantly higher among postgraduate laboratory staff and those who had more than 10 years of experience, which may reflect their better knowledge of vaccination importance and the grave consequences of not being vaccinated. In addition, there was significantly higher vaccination coverage among laboratory staff in private laboratories, which may reflect better biosafety practices and strict HBV vaccination requirements. Furthermore, laboratory staff who received the biosafety manual had higher vaccination coverage, which also reflects the influence of biosafety knowledge on vaccination.

In conclusion, occupational exposure to NSIs is still a major problem among laboratory staff in public and private laboratories in Sana’a, Yemen. The high incidence of NSIs among laboratory staff combined with not receiving first aid in nearly half of reported injuries increased the risk of HBV infection particularly among the nonvaccinated. Therefore, strengthening supervision, legalizing HBV vaccination for all laboratory staff, and optimizing laboratory practices regarding the management of sharps can minimize risks and prerequisites in Yemen.

## Abbreviations

HBV: hepatitis B virus
NSI: needle stick injury
